# Expression of Neural Markers by Undifferentiated Mesenchymal-Like Stem Cells from Different Sources

**DOI:** 10.1155/2014/987678

**Published:** 2014-03-05

**Authors:** Dana Foudah, Marianna Monfrini, Elisabetta Donzelli, Stefania Niada, Anna T. Brini, Monia Orciani, Giovanni Tredici, Mariarosaria Miloso

**Affiliations:** ^1^Department of Surgery and Translational Medicine, University of Milano-Bicocca, 20900 Monza, Italy; ^2^Department of Biomedical, Surgical and Dental Sciences, University of Milan, 20129 Milano, Italy; ^3^IRCCS Galeazzi Orthopaedic Institute, 20161 Milano, Italy; ^4^Department of Clinical and Molecular Sciences—Histology, University Politecnica delle Marche, 60126 Ancona, Italy

## Abstract

The spontaneous expression of neural markers, already demonstrated in bone marrow (BM) mesenchymal stem cells (MSCs), has been considered as evidence of the MSCs' predisposition to differentiate toward neural lineages, supporting their use in stem cell-based therapy for neural repair. In this study we have evaluated, by immunocytochemistry, immunoblotting, and flow cytometry experiments, the expression of neural markers in undifferentiated MSCs from different sources: human adipose stem cells (hASCs), human skin-derived mesenchymal stem cells (hS-MSCs), human periodontal ligament stem cells (hPDLSCs,) and human dental pulp stem cells (hDPSCs). Our results demonstrate that the neuronal markers **β**III-tubulin and NeuN, unlike other evaluated markers, are spontaneously expressed by a very high percentage of undifferentiated hASCs, hS-MSCs, hPDLSCs, and hDPSCs. Conversely, the neural progenitor marker nestin is expressed only by a high percentage of undifferentiated hPDLSCs and hDPSCs. Our results suggest that the expression of **β**III-tubulin and NeuN could be a common feature of stem cells and not exclusive to neuronal cells. This could result in a reassessment of the use of **β**III-tubulin and NeuN as the only evidence proving neuronal differentiation. Further studies will be necessary to elucidate the relevance of the spontaneous expression of these markers in stem cells.

## 1. Introduction

Mesenchymal stem cells (MSCs) from bone marrow (BM-MSCs) are the adult stem cells that have been best characterized but other similar populations have been isolated and described from several tissues such as adipose tissue, umbilical cord blood, skin, skeletal muscle, and teeth. When MSCs are isolated from various tissues, a heterogeneous pool of cells may be obtained, so it is crucial to distinguish MSCs from other cells that may exhibit a similar phenotype in culture [1]. In order to allow for an unequivocal isolation and identification of MSCs, a single marker or set of markers is required. In 2006 the International Society of Cellular Therapy established three minimal criteria for identifying MSCs and among these were the expression of the specific antigens CD73, CD90, and CD105 and the lack of the expression of CD34, CD45, and HLA-DR [2]. Subsequently, novel and alternative markers have been proposed which may be common or specific for MSCs derived from various sources [1]. Furthermore, marker expression is an important tool in order to prove MSC differentiation into different lineages.

We and other researchers have shown that undifferentiated BM-MSCs express neuronal and glial differentiation markers [3, 4]. This finding has been considered as evidence of the BM-MSCs' predisposition to differentiate toward neural lineages, thereby supporting the use of these cells in therapy for neurodegenerative diseases and other neurological disorders. Many clinical trials have been undertaken with BM-MSCs in neural repair [5] and, although promising evidence has been obtained, further studies are necessary. Some problems arise from the use of BM-MSCs because these cells are not very abundant, and their number and proliferative capacity decrease with the donor's age. Adipose stem cells (ASCs), dental stem cells (DSCs), and skin-derived mesenchymal stem cells (S-MSCs) represent a very interesting source of stem cells due to their great accessibility and availability and more rapid proliferative kinetics and greater expansion capabilities than BM-MSCs [6–8]. In order to verify whether there may be a possible use for these MSC-like cells in stem cell-based therapies for neurodegenerative diseases and nervous system injuries, it is essential to establish whether these stem cells, like BM-MSCs, spontaneously express neural markers. To this end, we evaluated by immunocytochemistry, immunoblotting, and fluorescence-activated cell sorting (FACS) experiments the expression of neuronal, glial, and neural progenitor markers at various culture passages in undifferentiated human ASCs (hASCs), human S-MSCs (hS-MSCs), human periodontal ligament stem cells (hPDLSCs), and human dental pulp stem cells (hDPSCs).

## 2. Materials and Methods

### 2.1. hASC Isolation and Characterization

hASC isolation and characterization were performed as previously described [9]. Briefly, subcutaneous adipose tissues were obtained from healthy patients undergoing plastic surgery after written consent and Institutional Review Board (IBR) authorization from the IRCCS Galeazzi Orthopaedic Institute of Milan. Raw lipoaspirates (15–150 mL) were enzymatically digested with 0.075% type I collagenase at 37°C under continuous agitation for 30 minutes. The stromal vascular fraction (SVF) was centrifuged (1200 g × 10 minutes) and filtered through a sterile medication lint and cells were plated (10^5^ cells/cm^2^) in DMEM (Sigma-Aldrich, Milan, Italy) supplemented with 50 U/mL penicillin, 50 *μ*g/mL streptomycin, and 2 mM L-glutamine (Sigma-Aldrich) (complete DMEM) plus 10% FBS. hASCs have been analysed for MSC-related cell surface antigens expression and for their differentiation ability toward cells of the mesodermal lineage [10]. For the experiments performed in this study, hASCs were maintained in complete DMEM plus 10% defined FBS in a 5% CO_2_ humidified incubator at 37°C.

### 2.2. hS-MSC Isolation and Characterization

hS-MSC isolation and characterization were performed as previously described [11, 12]. Skin biopsies of the mammary gland were obtained from healthy adult patients undergoing cosmetic plastic surgery after informed consent. In brief, the samples, with a size range between 10 and 15 mm, were postoperatively collected and rinsed twice with PBS supplemented with antibiotic-antimycotic solution (100 units/mL penicillin, 100 *μ*g/mL streptomycin, and 0.25 *μ*g/mL amphotericin B (Lonza, Verviers, Belgium). Under a stereo microscope, the fat tissue and most of the derma were carefully removed with forceps, and the remaining dermal-epidermal samples were finely shredded into smaller strips and then transferred into six-well plates with the culture medium Mesenchymal Stem Cell Growth Medium bullet kit (MSCGM) (Lonza, Verviers, Belgium). The cultures were maintained in MSCGM in a 5% CO_2_ humidified incubator at 37°C. After 14 days of culture, numerous cells forming colonies (CFU-F) migrated from the explants; nonadherent cells and residual dermal tissue were removed and the medium was replaced with a fresh one. Subsequently, the medium was changed twice a week. When the adherent cells reached confluence, they were detached by 0.125% trypsin and 1 mM ethylenediaminetetraacetic acid (EDTA) for 3 minutes. The released cells were collected and replated for subculturing in culture flasks with the same culture medium.

hS-MSCs expressed MSC-related cell surface antigens [12] and were able to differentiate toward mesodermal lineages [13]. For the experiments performed in this study, hS-MSCs were cultured in MSCGM in a 5% CO_2_ humidified incubator at 37°C.

### 2.3. hPDLSC and hDPSC Isolation and Characterization

Healthy human third molars extracted during orthodontic treatment were collected from patients, after obtaining written informed consent, at the Department of Dentistry, San Gerardo Hospital, Monza. All interventions were performed under anesthesia due to total mucosal or bone impaction of dental elements. After local anesthesia, performed in a plessic way in association with mepivacaine 2% with a 1 : 100000 adrenalin ratio, a full thickness flap was completed. Moreover, through osteotomy, impacted third molars were enucleated and then preserved for a maximum of 1 hour in a sterile saline solution (PBS). The following procedures were done under sterile conditions.

To obtain hPDLSCs, periodontal ligament tissue was gently scraped from the surface of the middle third of the root. The tissue was placed in a 75 cm^2^ culture flask with complete DMEM and incubated at 37°C in 5% CO_2_. To obtain hDPSCs, the teeth were sectioned longitudinally in a mesiodistal direction with a piezoelectric ultrasonic device (OT7 insert) under abundant irrigation with sterile salt solution (0.9% NaCl) to expose the pulp chamber. The pulp tissue was separated by means of an MOD.31W hand excavator; it was then placed in a 75 cm^2^ culture flask with complete DMEM plus 10% FBS and incubated at 37°C in 5% CO_2_.

hPDLSCs and hDPSCs expressed MSC-related cell surface antigens and were able to differentiate toward mesodermal lineages (paper in preparation).

For the experiments performed in this study, hPDLSCs and hDPSCs were cultured in complete DMEM plus 10% defined FBS in a 5% CO_2_ humidified incubator at 37°C.

### 2.4. Immunofluorescence Experiments

The expression of differentiation markers was determined in hASCs (P3, P6, and P16), hS-MSCs (P4, P8, and P16), hPDLSCs (P2, P4, and P8), and hDPSCs (P2, P4, and P8) by immunofluorescence experiments after 14 days from plating on glass slides in 35 mm diameter dishes (10^4^ cells/dish) in the culture medium used for cell expansion without adding any differentiative agent.

Cells were fixed with 4% paraformaldehyde for 10 min and then treated with 0.1 M glycine (Sigma-Aldrich, St. Louis, MO) for 10 min. Cells were incubated for 30 min at room temperature with a blocking solution (5% BSA, 0.6% Triton X-100 in PBS) and for 30 min at 37°C with 1 mg/mL RNAse (Sigma-Aldrich, St. Louis, MO). Incubation with the following primary antibodies was performed overnight at 4°C: mouse anti-human nestin (Chemicon, Temecula, CA; 1 : 50); rabbit anti-human nestin (Chemicon, Temecula, CA; 1 : 200); anti-*β*III-tubulin (Covance, Berkeley, CA; 1 : 100); anti-NeuN (Chemicon, Temecula, CA; 1 : 50); anti-Neurofilament (DakoCytomation, Glostrup, Denmark; 1 : 100); anti-GFAP (Sigma-Aldrich, St. Louis, MO; 1:100); anti-S100 (Chemicon, Temecula, CA; 1 : 100); mouse anti-osteopontin (Santa Cruz Biotechnology, Inc; 1 : 100); anti-osteocalcin (Abcam, Cambridge, UK; 1 : 100); anti-PPAR*γ*2 (Abcam, Cambridge, UK; 1:500). The following day, cells were washed with PBS plus 0.3% Triton X-100 and incubated for 1 hour in the dark with appropriate fluorochrome-conjugated secondary antibodies (Alexa Fluor 488, 555, 647 anti-mouse and anti-rabbit) (Invitrogen, Oregon, USA; 1 : 200). Propidium iodide (Sigma-Aldrich, St. Louis, MO; 2.5 *μ*g/mL) as a nuclear marker, or Alexa Fluor 546-conjugated phalloidin (Invitrogen, Oregon, USA; 1 : 200) as a cytoskeleton filamentous actin marker, was used. After incubation with the appropriate fluorochrome-conjugated secondary antibodies, cells were washed with PBS and mounted with polyvinyl alcohol. Microscopy analysis was performed with laser confocal microscopy (Radiance 2100; Biorad Laboratories, Hercules, CA, USA) and noise reduction was achieved by using Kalman filters during acquisition.

### 2.5. Cell Lysate and Immunoblotting Analysis

Total cellular extracts from hASCs, hS-MSCs, hPDLSCs, and hDPSCs were prepared as previously described [14]. To obtain nuclear protein extracts the Ronca et al. protocol was performed [15]. Protein concentration was determined by the Bradford assay using a Coomassie Protein Assay Reagent Kit (Pierce, Rockford, IL, USA). After electrophoresis onto 13% SDS-PAGE, the proteins were transferred to nitrocellulose filters and immunoblotting analysis was performed using anti-*β*III-tubulin (1 : 3000) and anti-NeuN (1 : 200) antibodies. Anti-actin (1 : 1000, Santa Cruz, Temecula, CA, USA) antibody was used as a loading control. After incubation with primary antibodies, the membrane was washed and then incubated with the appropriate horseradish peroxidise conjugated secondary antibodies (1 : 2000) (anti-mouse, Chemicon, Temecula, CA; anti-rabbit, PerkinElmer, Boston, MA; anti-goat, Santa Cruz Temecula, CA, USA) and the ECL chemiluminescence system (Amersham, Arlington Heights, IL, USA) was used.

### 2.6. Flow Cytometry Analysis

hASCs, hS-MSCs, hPDLSCs, and hDPSCs were trypsinized and centrifuged at 500 g for 5 min. Then, after washes with PBS, cells were fixed in 2% paraformaldehyde, permeabilized with 0.5% saponin for 15 min (for *β*III-tubulin staining), or fixed and permeabilized with cold methanol/acetone (3 : 1) for 30 min (for NeuN). Then cells were incubated with anti-*β*III-tubulin (1 : 100) or anti-NeuN (1 : 50) for 30 min at 4°C. After incubation with primary antibodies, cells were washed and then incubated with appropriate APC- or FITC-conjugated secondary antibodies for additional 30 min at room temperature. As control, cells were incubated only with the secondary antibody. At least 40,000 events were acquired with a cytometer (BD FACScantoI, BD Biosciences, San Jose, CA, USA) in a user-defined gate. Data were analyzed using FACS Diva software.

### 2.7. Statistical Analysis

Differences in the number (%) of cells expressing a specific differentiation marker in different passages were analyzed by using one-way analysis of variance (ANOVA). For each marker, an average value of positive cells after 14 days of culture was calculated. Data were expressed as means ± SD. Comparisons of mean values for the passages were analyzed using Tukey's multiple comparison test. A five percent probability (*P* < 0.05) was used as the level of significance.

## 3. Results 

### 3.1. Expression of Mesengenic and Neural Markers by Undifferentiated hASCs, hS-MSCs, hPDLSCs, and hDPSCs

At different culture passages and for each analysed passage at day 14 from plating we evaluated, in undifferentiated hASCs, hS-MSCs, hPDLSCs, and hDPSCs, and cultured, in the presence of serum and in the absence of any differentiative agent, the expression of the following differentiation markers: the neuronal markers of *β*III-tubulin [16], NeuN [17], and Neurofilament (NF) [18]; the glial markers of GFAP [19] and S100 [20]; the osteogenic markers of osteopontin (OPN) [21] and osteocalcin (OCN) [22]; the adipogenic marker PPAR*γ*2 [23]. Moreover, we analyzed the expression of the neuroprogenitor marker nestin [24]. For this purpose, we carried out immunofluorescence experiments and for each marker we obtained the percentage of positive cells by averaging experimental results. In each experiment, in ten randomly chosen microscopic fields, positive cells were counted and averaged. In all the experiments the markers retained their proper cellular localization.

#### 3.1.1. Undifferentiated hASCs

For each marker, the percentage of hASC positive cells was obtained by averaging the experimental results using cells from 3 healthy donors. The data reported in Table 1 refer to an average value (mean ± SD) for a positive marker after 14 days of culture at P3, P6, and P16.

**Figure 1:**
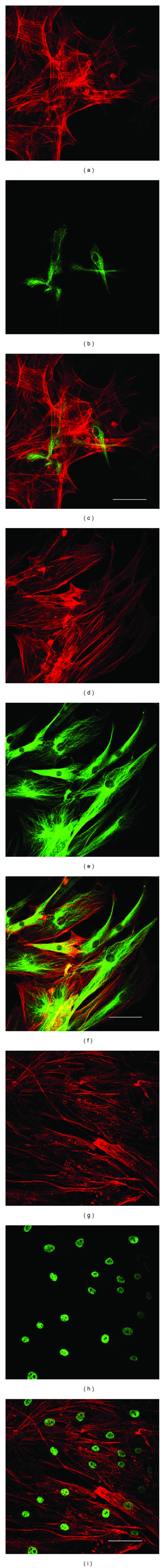
Spontaneous expression of neural markers by undifferentiated hASCs (P3), after 14 days of culture. Actin filaments were stained in red by phalloidin ((a), (d), and (g)), and neural markers were labelled in green. Few cells were nestin positive (b). Most of cells were *β*III-tubulin positive (e). Numerous cells were NeuN positive with nuclear localization (h). Merges ((c), (f), and (i)). Bars: 50 *μ*m.

A small number of undifferentiated hASCs expressed the neuroprogenitor marker nestin (Figure 1(b)). The expression of nestin was limited to about 1–5% of cells at P3 and P6, and at P16 no nestin positive cells were observed (Table 1).

As shown in Figure 1, undifferentiated hASCs expressed early and late neuronal markers. At all passages examined, more than 90% of undifferentiated hASCs resulted positive for *β*III-tubulin (an early neuronal marker) that showed a major concentration in perinuclear areas (Figure 1(e)), while NeuN (a late neuronal marker) was localized in the nucleus (Figure 1(h)) and expressed by about 60–80% of cells. The expression of the late neuronal marker NF was not observed at any culture passage examined. The expression of the glial marker GFAP was not observed at P3, while at P6 about 10% of cells were positive and at P16 this number increased to about 75%. Often GFAP expression was not equally distributed in the cytoplasm, and zones with different labelling intensities were observed. On the contrary, the expression of the glial marker S100 was not observed at any culture passage examined. Regarding the expression of mesengenic markers, undifferentiated hASCs did not express PPAR*γ*2 (adipogenic marker), OPN (early osteogenic marker), or OCN (late osteogenic marker) at any passage examined.

**Figure 2:**
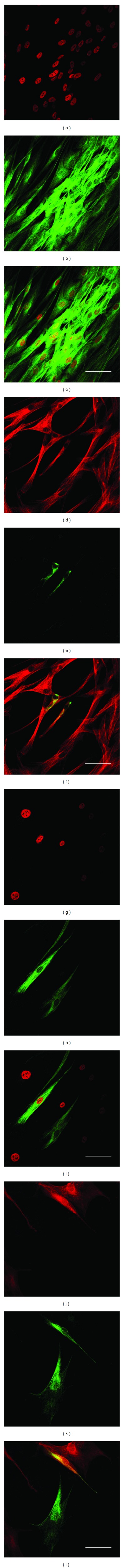
Spontaneous coexpression of neural markers by undifferentiated hASCs (P6) after 14 days of culture. NeuN-positive cells ((a): red) were always *β*III-tubulin positive ((b): green) as shown in merge (c). Most of cells were *β*III-tubulin positive ((d): red), and the few nestin-positive cells ((e), (h), and (k): green) were always *β*III-tubulin positive as shown in merge (f), while only in some case they were positive, respectively, for NeuN ((g): red) and GFAP ((j): red) as shown in merges (i) and (l). Bars: 50 *μ*m.

Moreover, double immunolabeling studies revealed that undifferentiated hASCs that were positive for NeuN also expressed *β*III-tubulin, while some *β*III-tubulin positive cells were NeuN negative (Figures 2(a)–2(c)). The percentage of nestin positive cells was very limited but these cells were always *β*III-tubulin positive (Figures 2(d)–2(f)) and, in some cases, they were, respectively, NeuN positive (Figures 2(g)–2(i)) and GFAP positive (Figures 2(j)–2(l)).

#### 3.1.2. Undifferentiated hS-MSCs

For each marker, the percentage of hS-MSC positive cells was obtained by averaging the experimental results using cells from 3 healthy donors. The data reported in Table 2 refer to an average value (mean ± SD) for a positive marker after 14 days of culture at P4, P8, and P16.

**Figure 3:**
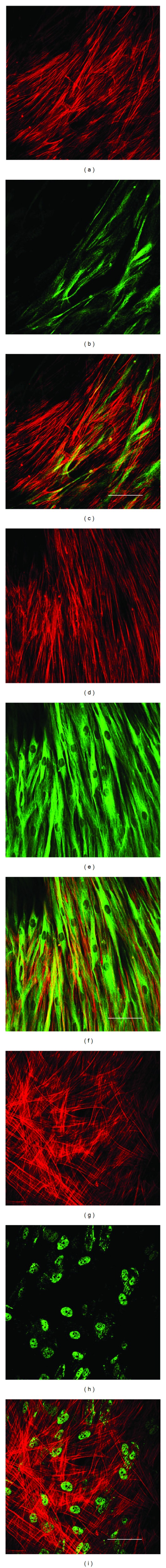
Spontaneous expression of neural markers by undifferentiated hS-MSCs (P4) after 14 days of culture. Actin filaments were stained in red by phalloidin ((a), (d), and (g)), and neural markers were labelled in green. Some cells were nestin positive (b). Most of the cells were *β*III-tubulin positive (e) and numerous cells were NeuN positive with nuclear localization (h). Merges ((c), (f), and (i)). Bars: 50 *μ*m.

The expression of the neuroprogenitor marker nestin was extended to about 10–15% of cells at P4 (Figure 3(b)). At P8, variability between donors was observed. At P16, on the other hand, for all donors examined, the number of cells expressing nestin decreased to about 1–3% (Table 2).

Undifferentiated hS-MSCs expressed the early neuronal marker *β*III-tubulin and at all passages examined more than 90% of cells resulted positive for this protein (Figure 3(e)). The late neuronal marker NeuN was expressed by about 80% of hS-MSCs (Figure 3(h)), at P4 and P8. At p16, on the other hand, variability between donors was observed NeuN being expressed by 80% of cells in two donors and by 5% of cells in one donor. The expression of the late neuronal marker NF was not observed at any culture passage examined.

The glial marker GFAP was not expressed at P4, while at P8 its expression was observed only in hS-MSCs deriving from one of the donors examined where about 30% of cells were GFAP positive. At P16, hS-MSCs obtained from two of the donors examined were GFAP positive (about 70%), while cells from the third donor were GFAP negative. Often GFAP expression was not equally distributed in the cytoplasm, and zones with different labelling intensities were observed.

Undifferentiated hS-MSCs did not express S100 (glial marker), PPAR*γ*2 (adipogenic marker), OPN (early osteogenic marker), or OCN (late osteogenic marker) at any passage examined.

**Figure 4:**
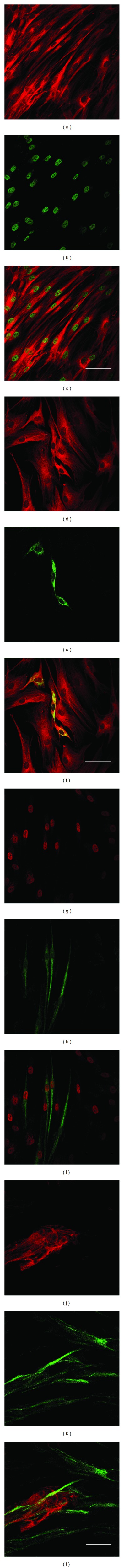
Spontaneous coexpression of neural markers by undifferentiated hS-MSCs (P8) after 14 days of culture. Most of cells were *β*III-tubulin positive ((a), (d): red) and the numerous NeuN-positive cells ((b): green) were always *β*III-tubulin positive as shown in merge (c). The few nestin-positive cells ((e), (h), and (k): green) were always *β*III-tubulin positive as shown in merge (f), while only in some cases they were positive, respectively, for NeuN ((g): red) and GFAP ((j): red) as shown in merges (i) and (l). Bars: 50 *μ*m.

Moreover, double immunolabeling studies revealed that undifferentiated hS-MSCs that were positive for NeuN also expressed *β*III-tubulin, while some *β*III-tubulin positive cells were NeuN negative (Figures 4(a)–4(c)). The percentage of nestin positive cells was very limited but these cells were always *β*III-tubulin positive (Figures 4(d)–4(f)) and, in some cases, they were, respectively, NeuN positive (Figures 4(g)–4(i)) and GFAP positive (Figures 4(j)–4(l)).

#### 3.1.3. Undifferentiated hPDLSCs

For each marker, the percentage of hPDLSC positive cells was obtained by averaging experimental results using cells from 3 healthy donors. The data reported in Table 3 refer to an average value (mean ± SD) for a positive marker after 14 days of culture at P2, P4, and P8.

**Figure 5:**
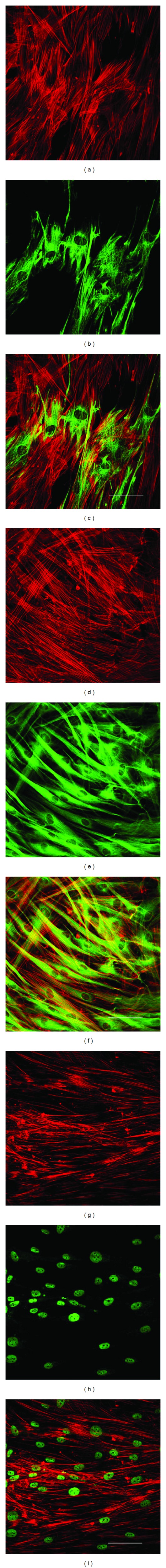
Spontaneous expression of neural markers by undifferentiated hPDLSCs (P4), after 14 days of culture. Actin filaments were stained in red ((a), (d), and (g)), and neural markers were labelled in green. About one-third of the cells in culture were nestin positive (b). Most of cells were *β*III-tubulin positive (e). Numerous cells were NeuN positive with nuclear localization (h). Merges ((c), (f), and (i)). Bars: 50 *μ*m.

At all the examined passages about 30% of undifferentiated hPDLSCs expressed the neuroprogenitor marker nestin (Figure 5(b)), while more than 90% of cells resulted positive for *β*III-tubulin (Figure 5(e)). NeuN expression showed variability between donors. At all the examined passages NeuN was expressed by 10% of cells in one donor and by 60–70% of cells derived from the other two donors (Figure 5(h)).

The expression of the late neuronal marker NF, the glial markers GFAP and S100, and the mesengenic markers PPAR*γ*2, OPN, and OCN was not observed at any culture passage examined.

**Figure 6:**
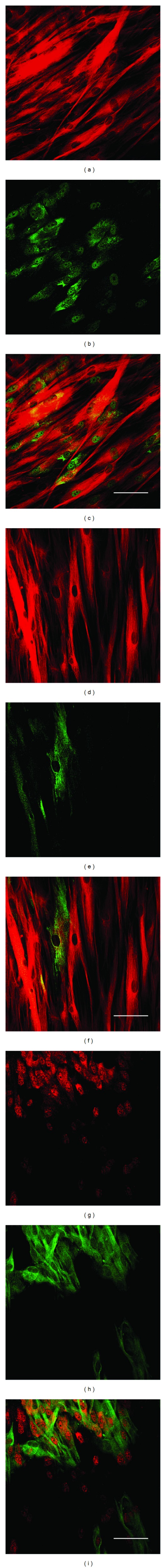
Spontaneous coexpression of neural markers by undifferentiated hPDLSCs (P8) after 14 days of culture. Most of cells were *β*III-tubulin positive ((a), (d): red) and the numerous NeuN-positive cells ((b): green) were always *β*III-tubulin positive as shown in merge (c). The nestin-positive cells ((e), (h): green) were always *β*III-tubulin positive as shown in merge (f), while only in some cases they were positive for NeuN ((g): red) as shown in merge (i). Bars: 50 *μ*m.

Moreover, undifferentiated hPDLSCs that were positive for NeuN also expressed *β*III-tubulin, while some *β*III-tubulin positive cells were NeuN negative, as demonstrated by double immunolabeling experiments (Figures 6(a)–6(c)). In hPDLSC cultures, nestin positive cells were always *β*III-tubulin positive (Figures 6(d)–6(f)) and, in some cases, they were also NeuN positive (Figures 6(g)–6(i)).

#### 3.1.4. Undifferentiated hDPSCs

For each marker, the percentage of hDPSC positive cells was obtained by averaging experimental results using cells from 3 healthy donors. The data reported in Table 4 refer to an average value (mean ± SD) for a positive marker after 14 days of culture at P2, P4, and P8.

**Figure 7:**
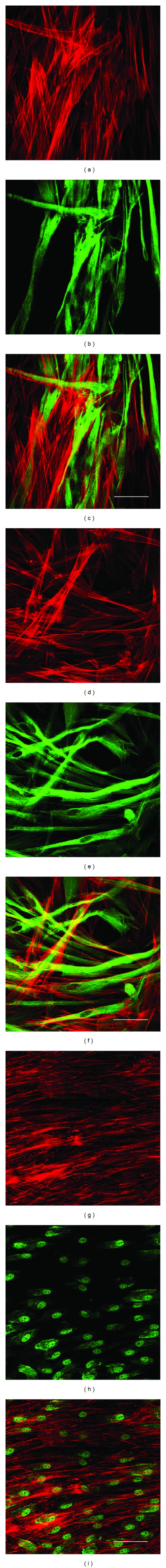
Spontaneous expression of neural markers by undifferentiated hDPSCs (P4), after 14 days of culture. Actin filaments were labelled in red ((a), (d), and (g)), and neural markers were labelled in green. About one-third of the cells in culture were nestin positive (b). Most of cells were *β*III-tubulin positive (e) and numerous cells were NeuN positive with nuclear localization (h). Merges ((c), (f), and (i)). Bars: 50 *μ*m.

At all the examined passages about 30% of undifferentiated hDPSCs expressed the neuroprogenitor marker nestin (Figure 7(b)), while more than 90% of cells were *β*III-tubulin positive (Figure 7(e)) and about 50–60% were NeuN positive (Figure 7(h)).

The expression of the late neuronal marker NF and the glial markers GFAP and S100 was not observed at any culture passage examined. Undifferentiated hDPSCs did not express PPAR*γ*2, OPN, and OCN at any passage examined.

**Figure 8:**
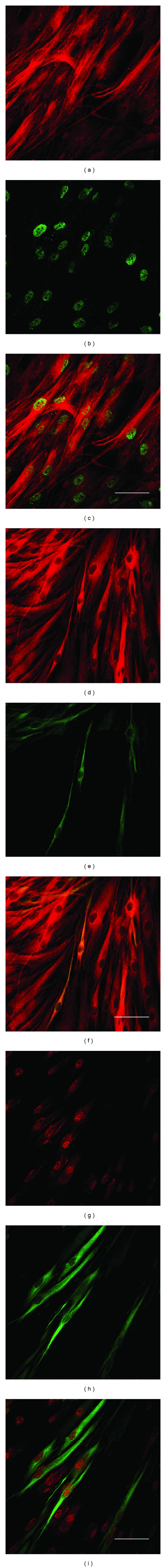
Spontaneous coexpression of neural markers by undifferentiated hDPSCs (P8) after 14 days of culture. Most of cells were *β*III-tubulin positive ((a), (d): red) and the numerous NeuN-positive cells ((b): green) were always *β*III-tubulin positive as shown in merge (c). The nestin-positive cells ((e), (h): green) were always *β*III-tubulin positive as shown in merge (f), while only in some cases they were positive for NeuN ((g): red) as shown in merge (i). Bars: 50 *μ*m.

Moreover, double immunolabeling studies revealed that undifferentiated hDPSCs that were positive for NeuN also expressed *β*III-tubulin, while some *β*III-tubulin positive cells were NeuN negative (Figures 8(a)–8(c)). In hDPSC cultures, nestin positive cells were always *β*III-tubulin positive (Figures 8(d)–8(f)) and, in some cases, they were also NeuN positive (Figures 8(g)–8(i)).

#### 3.1.5. Statistical Comparison among Different MSC Types

Among the neural markers evaluated, only *β*III-tubulin and NeuN were expressed by all the various undifferentiated mesenchymal-like stem cells from different sources. The overall means of the percentage of cells expressing *β*III-tubulin and NeuN for hASCs, hS-MSCs, hPDLSCs, and hDPSCs were calculated and a statistical comparison of these neuronal markers between the different MSC types studied showed no significant differences (Table 5).

### 3.2. Evaluation of *β*III-Tubulin and NeuN Expressions by Immunoblotting and Flow Cytometry

**Figure 9:**
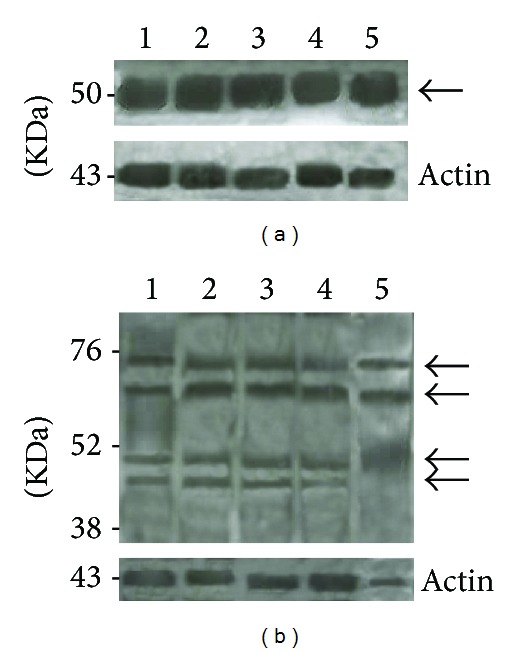
Stem cells expression of *β*III-tubulin and NeuN by immunoblotting. (a) Total protein extracts from hASC (1), hS-MSC (2), hPDLSC (3), hDPSC (4) and dorsal root ganglia (DRG) (5) cultures were separated by 13% SDS-PAGE and blotted with anti-*β*III-tubulin antibody. A 50 kDa molecular weight band was evident in the protein extracts from all the stem cells of different sources examined, as in positive control (DRG). Actin was used as loading control. (b). Nuclear protein extracts from hASC (1), hS-MSC (2), hPDLSC (3), hDPSC (4), and DRG (5) cultures were separated by 13% SDS-PAGE and blotted with anti-NeuN antibody. All the different types of stem cells examined express the two major NeuN species at 45–50 kDa, a reactive band at ~66 kDa, and another one between 70–90 kDa, with an expression profile similar to control. Actin was used as loading control.

**Figure 10:**
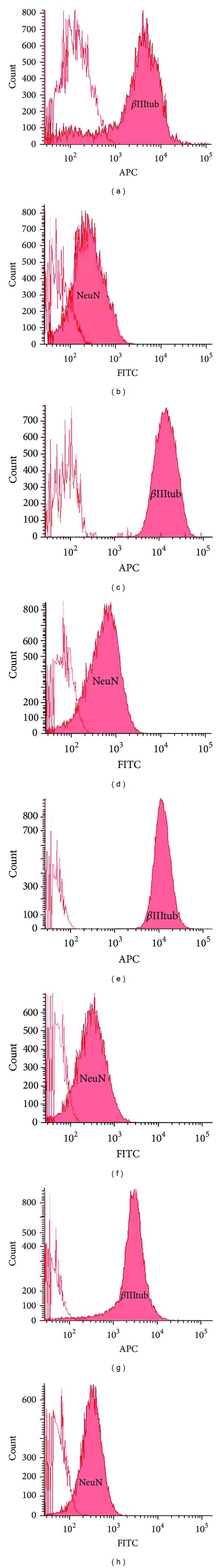
The undifferentiated stem cell expression of *β*III-tubulin and NeuN evaluated by flow cytometry analysis. *β*III-tubulin staining histograms (pink) of undifferentiated hASCs (a), hS-MSCs (c), hPDLSCs (e), and hDPSCs (g) and the respective negative controls represented by undifferentiated cells incubated with the appropriate APC-conjugated secondary antibody (white). NeuN staining histograms (pink) of undifferentiated hASCs (b), hS-MSCs (d), hPDLSCs (f), and hDPSCs (h) and the respective negative controls represented by undifferentiated cells incubated with the appropriate FITC-conjugated secondary antibody (white).

*β*III-tubulin and NeuN expressions in undifferentiated hASCs, hS-MSCs, hPDLSCs, and hDPSCs were evaluated through immunoblotting analysis. A band corresponding to a predicted 50 kDa molecular weight was evident in all the donor's total extracts examined (Figure 9(a)). As regards NeuN, the immunoblotting (nuclear extracts), showed two major bands at 45–50 kDa and additional reactive bands at ~66 kDa and between 70–90 kDa in accordance with the literature data [25] (Figure 9(b)). For all the stem cells of different origins examined, both *β*III-tubulin and NeuN positivity was confirmed by means of flow cytometric analysis, as shown in Figure 10.

## 4. Discussion and Conclusions

In this study, we have demonstrated that the early neuronal marker *β*III-tubulin and the late neuronal marker NeuN, unlike other evaluated markers, are expressed by a very high percentage of undifferentiated hASCs, hS-MSCs, hPDLSCs, and hDPSCs. These different types of stem cells spontaneously express the neuronal markers, in the absence of any differentiative agent, at all the passages examined. Other researchers have reported the expression of neural markers by hASCs and by stem cells from dental tissue [26–29], but no extensive studies with a panel of markers and culture passages such as ours have been published before.

Until now the expressions of *β*III-tubulin and NeuN have been considered to be limited to neuronal cells and have been used to prove *in vitro* neuronal differentiation. Our findings call into question these statements and raise the problem of identifying a role for these proteins in stem cells.

*β*III-tubulin is a constituent of neuronal microtubules and is required in axon growth/guidance and in normal brain development [16, 30]. However, the expression of *β*III-tubulin has been observed in cells other than neuronal ones such as tumor cells [31], perivascular cells (including pericytes and smooth muscle cells) [32], normal large intestine, fibroblasts, and keratinocytes [33]. Recently, alternative functions for *β*III-tubulin have been proposed. Shibazaki et al. [33] have demonstrated *β*III-tubulin involvement in the cell division of non-neuronal cells and Bouchet et al. [34] have shown that *β*III-tubulin is required for interphase microtubule dynamics in human mammary epithelial cells. Therefore, *β*III-tubulin could have different functions depending on the cell type. The transcriptional regulation of *β*III-tubulin represents another interesting aspect to be evaluated. REST/NRSF is a transcriptional regulator that binds to a highly conserved DNA sequence called RE1 located in many neuronal genes and that silences their transcription by recruiting specific corepressor multicomplexes [35]. In this way REST/NRSF represses the expression of mature neuron specific genes in non-neuronal cells and neuronal progenitors preventing neuronal differentiation [36]. Shibazaki et al. [33] have demonstrated that *β*III-tubulin expression is mediated by REST/NRSF and that the *β*III-tubulin level increases only in the G2/M phase when the occupancy in the RE-1 sequence is minimal. Conversely, in tumor cells, in which *β*III-tubulin is overexpressed, a dysregulation of REST/NRSF has been proposed [33].

In our work a very high percentage of hASCs, hS-MSCs, hPDLSCs, and hDPSCs are *β*III-tubulin positive suggesting that *β*III-tubulin expression could, therefore, be an intrinsic characteristic of these cells, probably being involved in their cell growth. A dysregulation of REST/NRSF or of their corepressors could explain why the level of *β*III-tubulin expression is so high. Further studies will be necessary to confirm this hypothesis.

NeuN is a neuron-specific nuclear protein and its expression is found only in postmitotic neurons [37]. Recently, NeuN has been identified as Rbfox3, a member of the RNA binding protein Fox-1 gene family [38]. NeuN/Rbfox3 regulates alternative splicing of Numb, a multifunctional protein expressed by a wide variety of cells and involved in many cellular processes including the maintenance of stem cell compartments [39]. The regulation of Numb by NeuN/Rbfox3 might not be restricted to neurons but extended also to stem cells. This hypothesis could explain why NeuN is expressed in all the MSC-like cells from the various sources examined in this study. Moreover, the importance of Numb has recently been demonstrated also in hDPSCs [40].

In our study, the percentage of cells expressing *β*III-tubulin and NeuN was quite similar in the various MSC-like cell types, although differences were evident regarding the nestin expression. While nestin was expressed by a high percentage of undifferentiated hPDLSCs and hDPSCs, only a small number of undifferentiated hASCs and hS-MSCs were nestin positive. The discrepancy observed in terms of the percentage of nestin expressing cells could be explained by taking into account the different origins of the stem cells examined in our study. hPDLSCs and hDPSCs originate from the neural crest [41] while for hASCs and hS-MSCs a mesoderm origin has been proposed [42]. Since nestin is a marker that is expressed not only by neural progenitors but also by neural crest cells [43], it is reasonable to assume that the high percentage of nestin positive hPDLSCs and hDPSCs reflects their embryonic origin.

An interesting aspect of our study is that none of the markers for mesodermal differentiation was spontaneously expressed by the various types of stem cells we looked at. This finding supports the hypothesis that the expression of the neuronal markers, observed at very high levels, is not aspecific but is probably related to the role played by these proteins in undifferentiated stem cells.

In a previous study, we showed *β*III-tubulin and NeuN expressions in undifferentiated human BM-MSCs [3] and the percentages of cells expressing these markers were similar to those observed for the MSC-like cells evaluated in this paper. Our previous and current findings suggest that the expressions of *β*III-tubulin and NeuN could be a common feature of stem cells, regardless of the tissue from which the cells are isolated. Moreover, the percentage of hBM-MSCs that were nestin positive was similar to the percentage reported for hASCs and hS-MSCS. Considering that BM-MSCs, like hASCs and hS-MSCs, originate from the mesoderm, in our opinion, this finding confirms that nestin expression by undifferentiated stem cells is closely related to their origin.

In conclusion, in this study we have demonstrated that, like hBM-MSCs, undifferentiated stem cells from various sources such as hASCs, hS-MSCs, hDPSCs, and hPDLSCs spontaneously express the neuronal markers *β*III-tubulin and NeuN. Further studies will be needed to analyze the gene expression of these markers to verify if a different regulation of these genes before and after differentiation could occur. A deeper knowledge of the biological properties of stem cells derived from different tissues would be very useful in view of their potential clinical application in cell therapy as an alternative to MSCs from bone marrow.

Last but not least, our results confirm that the evaluation of marker expression can never be used as the only evidence to prove the neuronal differentiation of MSC-like cells; morphological changes and functional properties should also be evaluated.

## Figures and Tables

**Table 1:** Undifferentiated hASC expression of differentiation markers at different passages after 14 days of culture. The number of positive cells for each marker is expressed as % ± SD (—: no positive cells).

Marker	Passage			Significant differences (*P* value < 0.05, ANOVA)
	P3	P6	P16	
Nestin	5 ± 2.5	1.5 ± 0.5	—	All ns
*β*III-tubulin	90 ± 5	90.67 ± 3.06	91.67 ± 3.06	All ns
NeuN	75 ± 5	65 ± 5	65 ± 5	All ns
GFAP	—	10 ± 3.54	75 ± 7.07	P6 versus P16

**Table 2:** Undifferentiated hS-MSC expression of differentiation markers at different passages after 14 days of culture. The number of positive cells for each marker is expressed as % ± SD (—: no positive cells). When the values are very heterogeneous we report the % of positive cells for each donor.

Marker	Passage			Significant differences (*P* value < 0.05, ANOVA)
	P4	P8	P16	
Nestin	12.33 ± 2.52	@	3.17 ± 1.76	All ns
*β*III-tubulin	85 ± 5	90.67 ± 1.53	91.67 ± 0.58	All ns
NeuN	78.33 ± 7.64	75 ± 5	∞	All ns
GFAP	—	#	∗	All ns

^@^Donor 1: 2%; donor 2: 22%; donor 3: 7%.

^*∞*^Donor 1: 80%; donor 2: 5%; donor 3: 78%.

^#^Donor 1 and donor 2: negative; donor 3: 30%.

*Donor 1: negative; donor 2 and donor 3: 70%.

**Table 3:** Undifferentiated hPDLSC expression of differentiation markers at different passages after 14 days of culture. The number of positive cells for each marker is expressed as % ± SD.

Marker	Passage			Significant differences (*P* value < 0.05, ANOVA)
	P2	P4	P8	
Nestin	30 ± 5	31.67 ± 14.43	33.33 ± 11.55	All ns
*β*III-tubulin	90 ± 5	90.67 ± 3.06	91.33 ± 1.53	All ns
NeuN	50 ± 34.64	40 ± 26.46	41.67 ± 28.43	All ns

**Table 4:** Undifferentiated hDPSC expression of differentiation markers at different passages after 14 days of culture. The number of positive cells for each marker is expressed as % ± SD.

Marker	Passage			Significant differences (*P* value < 0.05, ANOVA)
	P2	P4	P8	
Nestin	33.33 ± 11.55	35 ± 8.66	25 ± 8.66	All ns
*β*III-tubulin	92.67 ± 2.52	91.67 ± 1.53	90 ± 3.61	All ns
NeuN	61.67 ± 12.58	53.33 ± 5.77	58.33 ± 10.41	All ns

**Table 5:** Overall means of the percentage of cells expressing *β*III-tubulin and NeuN in hASCs, hS-MSCs, hPDLSCs, and hDPSCs after 14 days of culture. The number of positive cells for each marker is expressed as % ± SD.

Marker	MSC type				Significant differences (*P* value < 0.05, ANOVA)
	hASCs	hS-MSCs	hPDLSCs	hDPSCs	
*β*III-tubulin	90.78 ± 3.4	89.11 ± 4.1	91.11 ± 3.3	91.44 ± 2.6	All ns
NeuN	68.33 ± 6.6	69.22 ± 24.6	43.89 ± 26.43	57.78 ± 9.39	All ns
